# Absence of *PITX3* mutation in a Tunisian family with congenital cataract and mental retardation

**Published:** 2010-04-03

**Authors:** Manèl Chograni, Myriam Chaabouni, Imen Chelly, Mohamed Bechir Helayem, Habiba Chaabouni-Bouhamed

**Affiliations:** 1 Faculté de Médecine de Tunis, Laboratoire Génétique Humaine, Tunis, Tunisia; 2 Charles Nicolle hospital, Congenital and Heredotary Disorders Department, Tunis, Tunisia; 3 Razi hospital, Child and Adolescent Psychiatry Department, La Manouba, Tunisia

## Abstract

**Purpose:**

The *PITX3 *(pituitary homeobox 3) gene encodes for a homeobox bicoid-like transcription factor. When one allele is mutated, it leads to dominant cataract and anterior segment mesenchymal dysgenesis in humans. When both copies are mutated, homozygous mutation contributes to microphtalmia with brain malformations. In the current study, a family with autosomal recessive congenital cataract (ARCC) associated with mental retardation (MR) was examined to identify *PITX3* mutations.

**Methods:**

Sequencing of the *PITX3* gene was performed on two affected and three unaffected members of the studied Tunisian family. The results were analyzed with Sequencing Analysis 5.2 and SeqScape.

**Results:**

No mutation in the four exons of *PITX3* was revealed. Two substitution polymorphisms, c.439C>T and c.930C>A, were detected in exons 3 and 4, respectively. These alterations did not segregate with the disease.

**Conclusions:**

Although *PITX3* was shown to be essential to normal embryonic eye and brain development in vertebrates, we report the absence of *PITX3* mutations in a family presenting congenital cataract and mental retardation.

## Introduction

Congenital cataracts occur in 30 of each 100,000 births [1]. They are most commonly inherited as autosomal dominant traits and have a variety of phenotypic expression [2].

Autosomal recessive congenital cataract (ARCC) has been assigned to a minority of genes in contrast to the autosomal dominant (ADCC) trait [3,4]. The *PITX3 *(pituitary homeobox 3)**gene (10q24.32) has been described among the wide set of genes responsible for ADCC but not for ARCC [5]. *PITX3* comprises four exons (exon 1=UTR [untranslated region]) and encodes a protein of 302 amino acid residues, which is expressed in the developing lens [6], skeletal muscle, and dopaminergic neurons [7] of the substantia nigra in the brain.

Human patients with point mutations in *PITX3* demonstrate congenital cataracts along with anterior segment defects in some cases when one allele is affected and microphthalmia with brain malformations when both copies are mutated [8]. Recently Bidinost et al. [8] reported a three-generation Lebanese family containing 28 affected members with posterior polar cataracts (PPCs) who were heterozygous for the *PITX3* mutation 650delG of exon 4; only two siblings from a consanguineous mating had microphtalmia with significant neurologic impairment and were homozygous for the deletion. Taking these results further, we analyzed the *PITX3* gene in a Tunisian family with ARCC associated to mental retardation (MR).

## Methods

### Subjects and sample collection

Five members of a family with congenital cataract associated with MR were enrolled in a genetic research program in the laboratory of Human Genetics, Faculty of Medicine Tunis, Tunisia. The family was referred to the Congenital and Hereditary Disorders Department at Charles Nicolle Hospital (Tunis, Tunisia) because of two affected daughters with ARCC and MR.

The two patients, aged 27 and 16 years were followed by ophthalmologists because of cataract clinically suspected since birth. They were examined at ages 4 and 6 years. The cataract was bilateral and of the posterior polar type, with white dots in the anterior vitreous and strabismus of the left eye for the younger sister. They had undergone an extracapsular extraction and implantation of the posterior chamber for both eyes. For the older sister, neurologic examination at age 21 years showed mild axial hypotonia and a mild spastic walk. The younger sister was examined again at the age of 13 years; neurologic examination showed mild axial hypotonia, increased tone and contracture in the lower limbs with flexed knees, tetrapyramidal syndrome, and persistent strabismus of the left eye. Muscle electromyography was normal.

**Figure 1:**
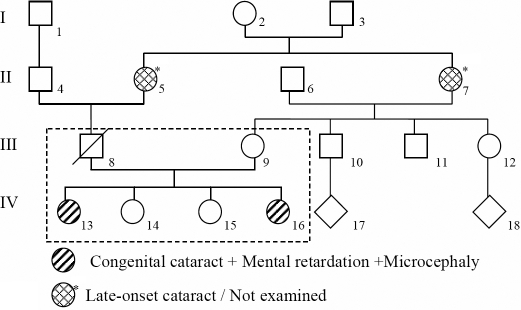
Pedigree of the Tunisian family showing patients IV:13 and IV:16 with congenital cataract associated with mental retardation and microcephaly. The pedigree of this family proved the autosomal recessive inheritance of congenital cataract. The asterisk indicates not examined.

The two sisters had microcephaly, absence of dysmorphic features, and normal menarche. They also had a significant delay in speech development and moderate mental retardation with learning and reading disability (Table 1). Neither of the two sisters had glaucoma before or after the extraction of cataract. They showed no signs of neurologic deterioration over the past decade. The patients were born from healthy consanguineous parents; the father had died from a traumatic accident. We noted that their grandmothers (II:5 and II:7) had late-onset cataract (Figure 1).

**Table 1:** Clinical characterestics of the two affected patients.

**Clinical features**	**Patient 1 (IV:13)**	**Patient 2 (IV:16)**
Age (1st consultation)	4 years	6 years
Weight	Normal	Normal
Height	Normal	Normal
Microcephaly	Present (−5.5 SD)	Present (−6.7 SD)
Cataract	Unilateral	Bilateral
Mental retardation	Moderate	Moderate
Retinal dystrophy	Absent	Present

Karyotyping with R-banding revealed normal karyotype 46,XX (600-band resolution) and normal metabolic screening including normal Fehling reaction and normal thin layer chromatography of reducing sugars,  normal plasmatic amino acid and urine organic acid chromatography. Magnetic resonance imaging of the brains was normal, except for the presence of a small ischemic parietal lesion in the younger sister.

We considered that this phenotype—cataract, microcephaly, and mental retardation—was concordant with autosomal recessive inheritance. Peripheral blood samples were taken from the two affected patients, two healthy sisters, and their mother and stored in 10 ml Vacuum tube sterile containing 100 µl of 0.1 M EDTA.K3. Blood samples were kept at room temperature for a short time before use. Informed consent was obtained from the healthy individuals and the parents of the affected patients. Genomic DNA was extracted from blood leukocytes by the standard proteinase-K extraction consisting on: lysis of red blood cells by RBC (Red Blood Cells) Lysis Buffer (155 mM NH4Cl, 10 mM KHCO3, 0.5 EDTA, pH 7.5) and white blood cells by a WBC (White Blood Cells) Lysis Buffer (1 mM Na-EDTA, 5mM Tris HCl pH 7.5), treatment of the lysate with a mixture of detergent composed of SDS (Sodium Dodecyl Sulfate or sacrosyl and proteinase K) in order to liberate the DNA and digest the associated proteins, precipitation of the DNA in the form of filaments by absolute ethanol and finally dilution of the DNA in T10E1 Buffer (Tris 10 mM, EDTA 0.1-1 mM).

### Molecular analysis

PCR primers of the exons and intron–exon junctions of all four coding exons of *PITX3* were designed according to Primer 3 and to amplify and to sequence a 1.3-Kb region, including the 5′-UTR upstream of the exon 1 translation start site and the 3′-UTR of exon 4. Their sequences are shown in Table 2 [8]. The gene sequence was retrieved from the Ensembl database (Ensembl protein_coding Gene: ENSG00000107859 [HGNC Symbol: PITX3]). The amplification reactions were performed by using 400 ng of DNA template, 20 pmol each of forward and reverse primers (Biomatik, Canada), 1.5 Units of Taq (*Thermus aquaticus*) DNA polymerase recombinant (Invitrogen, Carlsbad, CA) and 1.25 mM dNTPs (Promega, Madison, WI) in a total volume of 50 μl. Amplification was performed under the following conditions: 95 °C for 5 min, 95 °C for 30 s, then 54–60 °C for 30 s, and elongation at 72 °C for 30 s, followed by one cycle of final extension at 72 °C for 7 min, in a thermal cycle GeneAmp  PCR system 9700 (Applied Biosystems, Foster city, CA). The amplified products were purified (Wizard® SV Gel and PCR Clean-Up System Kit; Promega) and sequenced (Big Dye Terminator Cycle Sequencing Ready Reaction; DNA Sequencing Kit; ABI PRISM 3130; Applied Biosystems) in the forward and reverse directions. Sequencing results were visualized and data were computer analyzed using Sequencing Analysis 5.2 and SeqScape software (Applied Biosystems).

**Table 2:** Primers for *PITX3* and the flanking regions (UTR refers to non-translated region).

**Primer**	**Sequence**
Exon 1-F	5′-CCCTGGTCTGCCATAAAGTG-3′
Exon 1-R	5′-TTTAGGGATTCCAAGGGTCCA-3′
Exon 2-F	5′-GGCTGGGGTTGAGAAAGGCG-3′
Exon 2-R	5′-CCACTCGCTGGCTCCCACC-3′
Exon 3-F	5′-GCAGCCCCGGTGGGAGC-3′
Exon 3-R	5′-GGGAGGGGGCAGGTGGG-3′
Exon 4-F	5′-CCGTCTCTAGCCACCTCATC-3′
Exon 4-R	5′-CCAGTCAAAATGACCCCAGT-3′
Upstream distal-F	5′-AAGTCAGAGAGGGCCGAAGT-3′
Upstream distal-R	5′-CCAAGTGGGCGAGAGTAGAG-3′
Upstream proximal-F	5′-ATCCACTTTCCTCGGGGTAG-3′
Upstream proximal-R	5′-ACAGGCAGACTCCCAGTAGC-3′
3′ UTR-F	5′-CAACCTTAGTCCGTGCCAGT-3′
3′ UTR-R	5′-GAAGAGGACTCAAGCGCAAC-3′

## Results

Mutation screening of *PITX3* for the two affected siblings with the common phenotype of congenital cataract associated with MR did not reveal any mutation in the four exons of *PITX3*. Sequence analysis of the affected siblings, their normal sisters, and their mother showed substitution polymorphisms (c.439C>T and c.930C>A; we considered the first nucleotide of the cDNA sequence nucleotide number one), in exons 3 and 4, respectively, that were not included in a conserved domain of *PITX3* and did not segregate with the disease. The gene modifications were transmitted from the mother (heterozygous) to one affected child (heterozygous) for c.439C>T and from the mother to two affected children and one normal child for c.930C>A.

## Discussion

*PITX3* is described among the wide set of genes included in ADCC. It is responsible for the early development of the anterior segment of the eye [1,9]. The mouse homologue *Pitx3* was identified in 1997 and demonstrates strong expression during lens and brain development. It is expressed in tissues fated to contribute to eye development–the presumptive lens ectoderm, placode, and lens–and is involved in homozygous mice aphakia [10]. It was demonstrated in the frog (*Xenopus laevis*) [11] and zebrafish (*Danio rerio*) [12] that, contradictory to assertions that retina can form in the absence of lens, the expression of *Pitx3* in the presumptive lens ectoderm is critical for retinal development. In fact, the mutation in *PITX3*, a transcription factor containing a homeodomain, has been demonstrated to cause cataracts [5] and anterior segment mesenchymal dysgenesis [13] in several families from different ethnic origins.

The *PITX3* gene has been described in ADCC but not in ARCC. Recently, Bidinost and co-workers [8] reported one large Lebanese family where patients with a heterozygous mutation in *PITX3* (650delG) had PPCs, while patients with the same mutation but with the homozygous state had a more severe ocular effect with severe microphtalmia associated with developmental delay and mental retardation. In addition, the two homozygous brothers are offspring of a consanguineous marriage, and their parents, who presented with PPCs, were examined through the 28 affected members and were heterozygous for the reported deletion.

Despite the fact that *PITX3* is not a candidate gene for ARCC, we analyzed it because, first, it plays a crucial role in maintaining normal development for the eye and the brain [6] and second, this homeodomain transcription factor was found to be responsible for human congenital cataract and led to neurologic impairments, including MR [8]. Moreover, microphthalmia could be associated with ARCC, MR, and microcephaly in other syndromes, as the microsyndrome [14]. In this case the gene leading to severe microphthalmia should be expressed in the eye and the brain. Based on these observations, we studied the *PITX3* gene in a Tunisian family whose two affected members presented ARCC associated with MR and microcephaly but we did not identify any pathogenic mutation.

Our analyses have identified an already reported silent polymorphism, c.439C>T single nucleotide polymorphism (SNP rs2281983), in exon 3 that conserved the same amino acid Isoleucine (I) in the protein sequence p.I95I and did not segregate with the studied phenotype (association between ARCC, MR, and microcephaly) because of the heterozygosity of one affected child (IV:13). Another unreported variation (c.930C>A) in exon 4, resulting in a proline-to-leucine subtitution at position 258 of the protein, did not segregate with the phenotype because of the heterozygosity of the two affected children.

Our report confirms that *PITX3* is not involved in ARCC. Although the Lebanese-reported family and the Tunisian-studied family probably have the same Arabic origin, we did not identify a mutation or a polymorphism in *PITX3* that co-segregates with the studied phenotye (association between ARCC and MR). A genome-wide scan must be performed for this family to identify candidate genes.
